# Ag Nanoparticles Located on Three-Dimensional Pine Tree-Like Hierarchical TiO_2_ Nanotube Array Films as High-Efficiency Plasmonic Photocatalysts

**DOI:** 10.1186/s11671-017-1834-1

**Published:** 2017-01-19

**Authors:** Jinxia Xu, Zhenhuan Wang, Wenqing Li, Xingang Zhang, Dong He, Xiangheng Xiao

**Affiliations:** 1 Department of Physics and Key Laboratory of Artificial Micro- and Nano-Structures of Ministryof Education and Laboratory of Printable Functional Nanomaterials, Wuhan University, Wuhan, 430072 People’s Republic of China; 2 Hubei Collaborative Innovation Center for High-efficiency Utilization of Solar Energy and School of Electrical & Electronic Engineering, Hubei University of Technology, Wuhan, 430068 People’s Republic of China; 3 Su Zhou Institute of Wuhan University, Suzhou, 215123 People’s Republic of China

**Keywords:** Three-dimensional TiO_2_, Ag nanoparticles, Plasmonics, Photocatalysts

## Abstract

High specific surface area three-dimensional pine tree-like hierarchical TiO_2_ nanotube array films loaded with Ag nanoparticles were successfully prepared by one-step hydrothermal reaction combining with simple and feasible magnetron sputtering. The composite Ag/TiO_2_-branched nanotube arrays show outstanding photocatalytic property, which is attributed to the boost of plasmonic enhancement carrier generation and separation, higher specific surface area, higher organic pollutant absorption, faster charge transport, and superior light-harvesting efficiency for efficient charge collection. The work provides a cost-effective and flexible pathway to develop high-performance photocatalyst or optoelectronic devices.

## Background

In spite of nearly half a century investigations, since Fujishima and Honda discovered the photocatalytic water splitting on TiO_2_ electrodes in 1972 [1], TiO_2_ still remains to be intensively investigated as semiconductor photocatalyst owing to its important applications in phtocatalysis [2–7], photoelectrochemical water splitting [8–12], solar cell [13–16], and sensors [17, 18], because of its excellent chemical stability, abundance, and low cost. However, the photocatalytic activities of TiO_2_ are restricted by its low photocatalytic sensitivity in the UV region and rapid recombination of photogenerated electron and hole pairs [19]. Much effort has been dedicated to enhance the photocatalytic efficiency of TiO_2_ in the aspect of morphology, surface area, and surface defects. Zero-dimensional (D) TiO_2_ particles fabricated with randomly organized provides a large specific surface area for absorbing sufficient dye molecules. However, it has high charge recombination because of the large grain boundary of nanoparticles (NPs) [20, 21]. Then, well-aligned one-dimensional nanostructure such as nanowires, nanorods, and nanotubes were fabricated to improve charge transport due to a direct transport pathway for photogenerated electrons [22–24]. Nevertheless, the drawback of the low surface-to-volume ratio of one-dimensional nanostructure results to a low photocatalytic activity. Recently, researchers have been enthusiastically dedicated to develop three-dimensional nanostructures such as nanoflowers and nanotrees for application in photocatalysis [25–28]. Compared with zero-dimensional NPs and one-dimensional nanowires, the three-dimensional nanostructure offers the advantage of a large surface area that increases dye loading. Additionally, the three-dimensional morphology could offer long optical paths for efficient light absorption and abundant active sites for electrochemical reactions, providing efficient transport pathway for rapid charge transport that leads to improving electron collection and electron-hole separation.

TiO_2_ decorated with noble metal (Au, Ag, Pt, etc.) NPs named plasmonic photocatalysis is another promising method for enhancing the photocatalytic activity of TiO_2_ owing to the localized surface plasmon resonance (LSPR) effect of metal NPs [29–33]. The band gap of TiO_2_ is about 3.2 eV, and Ag NPs show a very strong LSPR absorption in the near-UV region [34, 35]. So, Ag is an optimal choice due to the Ag NPs’ LSPR position close to the exciton absorption of TiO_2_; on the other hand, silver is most suitable for industrial applications owing to its easy preparation and low cost. Moreover, the Ag NPs decorated on the surface of TiO_2_ could act as an electron trap center to effectively prevent electron-hole recombination and enhance the photocatalytic activity.

**Fig. 1:**
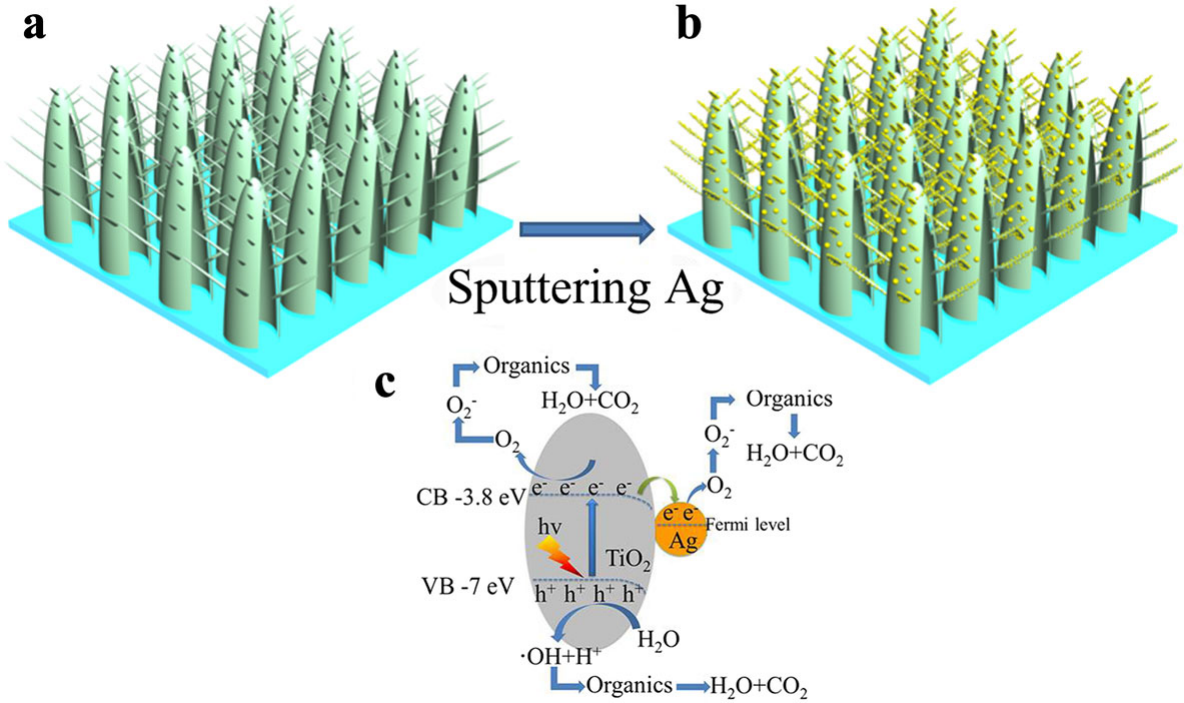
**a**, **b** Schematic illustration of the formation of Ag-decorated three-dimensional pine tree-like hierarchical TiO_2_ nanotube arrays. **c** Schematic illustration of the photogenerated electron transfer process in Ag/TiO_2_ comoposite under UV and visible light irradiation

Herein, we report a high-performance plasmonic photocatalyst three-dimensional pine tree-like hierarchical TiO_2_ nanotube array films loaded with Ag NPs fabricated by a simple two-step process. The composite Ag/TiO_2_-branched nanotube arrays show outstanding photocatalytic property, which is attributed to the boost of plasmonic-enhanced carrier generation and separation, higher specific surface area, higher organic pollutant absorption, faster charge transport, and superior light-harvesting efficiency for efficient charge collection. Figure 1 illustrates the formation procedure and proposed photocatalytic mechanism of three-dimensional pine tree-like hierarchical TiO_2_ nanotube array films loaded with Ag NPs. Compared with our previous work, the photocatalytic activity of three-dimensional pine tree-like hierarchical TiO_2_ nanotube array films loaded with Ag NPs was further enhanced.

## Results and Discussion

**Fig. 2:**
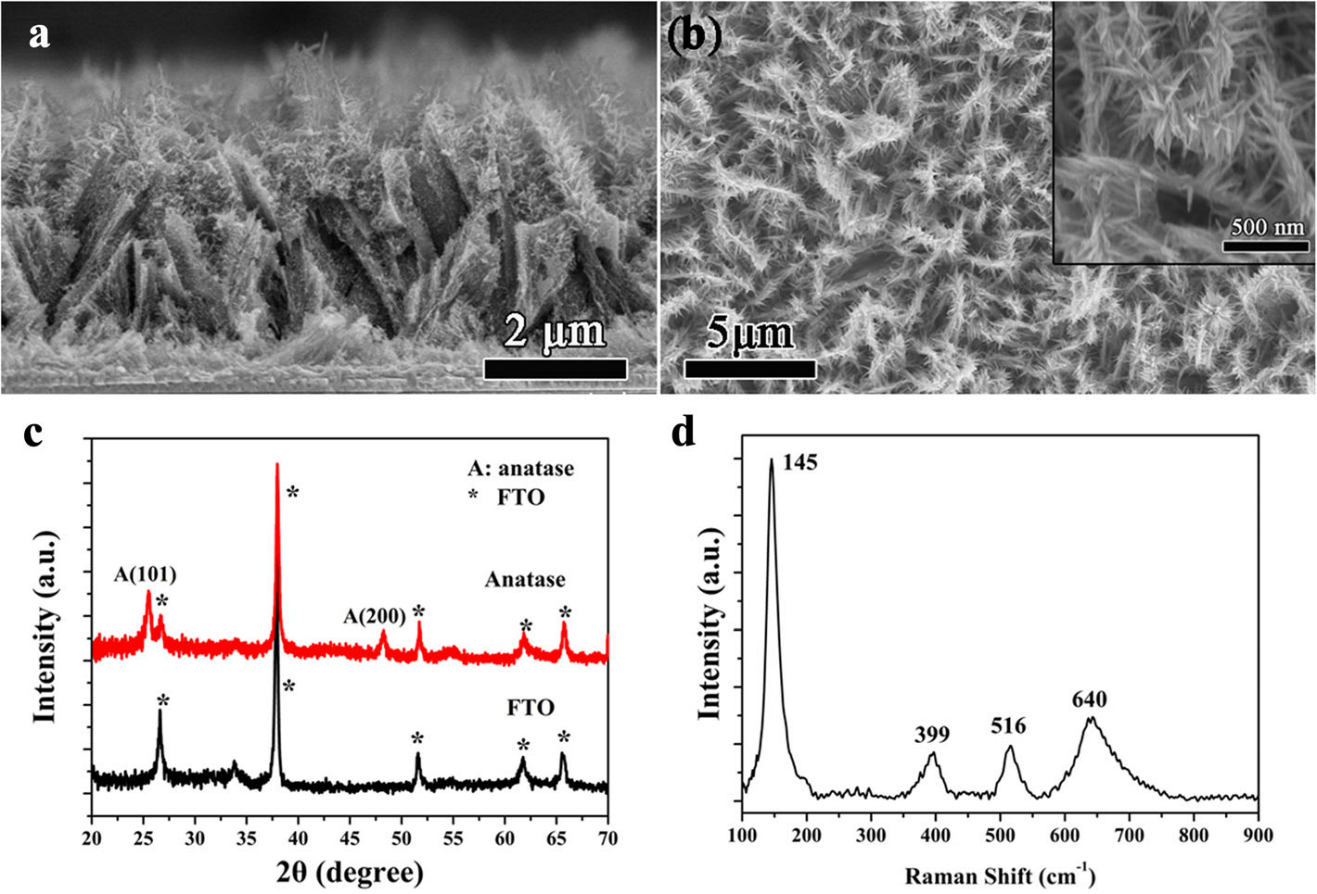
**a** Cross-sectional SEM image, **b** SEM image and magnified image (the inset), **c** XRD pattern, and **d** Raman spectrum of three-dimensional pine tree-like hierarchical TiO_2_ nanotube arrays

The microstructural and morphology detail of the prepared three-dimensional pine tree-like hierarchical TiO_2_ nanotube arrays is shown in Fig. 2a. The cross-sectional SEM images show that the prepared three-dimensional pine tree-like hierarchical TiO_2_ nanotube arrays composing of a vertically oriented nanotube trunk with a length of approximately 5 μm grafted with large amounts of short branches with lengths of about 300 nm were directly grown on FTO substrate by a simple one-step hydrothermal method. As shown in Fig. 2b the SEM image and magnified image (the inset) of three-dimensional pine tree-like hierarchical TiO_2_ nanotube arrays, the three-dimensional TiO_2_ nanotube arrays were fully covered and arranged homogeneously on the FTO glasses with large-scale and uniform growth and large amounts of short nanorod branches. The magnified image shows the diameter of the branch at approximately 50 nm. This hierarchical architecture with large specific surface area can enhance the absorption of dye molecules and effectively improve charge transport by a direct transport path thereby may be improving the photocatalytic activity of the TiO_2_. The phase purity and structure of the three-dimensional pine tree-like hierarchical TiO_2_ nanotube arrays were analyzed using XRD as shown in Fig. 2c. It can be found that diffraction peaks appeared at 25.4° and 48°, which can be attributed to the (101) and (200) orientations of the anatase TiO_2_ (JCPDS No.21-1272) [36]. No characteristic peak of any impurity is probed, which demonstrates that the sample fabricated by this method has high phase purity. Raman scattering as a local probe is very sensitive to microstructures and crystallinity of materials. Figure 2d shows the Raman scattering spectra of three-dimensional pine tree-like hierarchical TiO_2_ nanotube arrays. The founded Raman bands at 145, 399, 516, and 640 cm^−1^ can be separately indexed to the Eg, B1g, A1g, or B1g and Eg, which are consistent with the anatase phase of TiO_2_ and without any signal of a brookite or rutile phase [37, 38]. This result is consistent with the XRD. All the results show that the three-dimensional pine tree-like hierarchical anatase TiO_2_ nanotube array films were successfully fabricated.

**Fig. 3:**
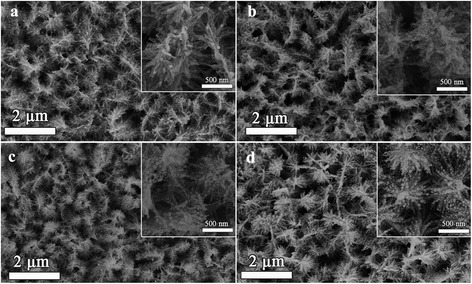
SEM images of three-dimensional pine tree-like hierarchical TiO_2_ nanotube arrays deposited with Ag by different deposition times: **a** 10 s, **b** 20 s, **c** 30 s, and **d** 40 s, and the set one is the corresponding magnified image

The typical SEM views of Ag NPs with deposition time of 10, 20, 30, and 40 s are shown in Fig. 3a–d, and the inset one is the corresponding magnified image. It can be observed that Ag NPs are uniformly coated on the branches of TiO_2_. The mean diameter of Ag NPs is approximately13, 25, 35, and 45 nm in sample Ag(10s)/TiO_2_, Ag(20s)/TiO_2_, Ag(30s)/TiO_2_, and Ag(40s)/TiO_2_, respectively. It is obvious that the diameter of Ag NPs increases with the increase of silver deposition time. So, Ag NPs deposited uniformly on three-dimensional pine tree-like hierarchical TiO_2_ nanotube arrays by a simple magnetron sputtering system were fabricated. It is well know that the LSPR effect of Ag NPs can form a strong local electronic field. Moreover, Ag NPs here were decorated uniformly on the branches of TiO_2_, so the near-field dipolar interactions between adjacent particles [39] were very strong. Therefore, it may form a stronger local electronic field in the hierarchical structure. So, Ag NPs deposited on three-dimensional pine tree-like hierarchical TiO_2_ nanotube arrays are expected with higher performance.

**Fig. 4:**
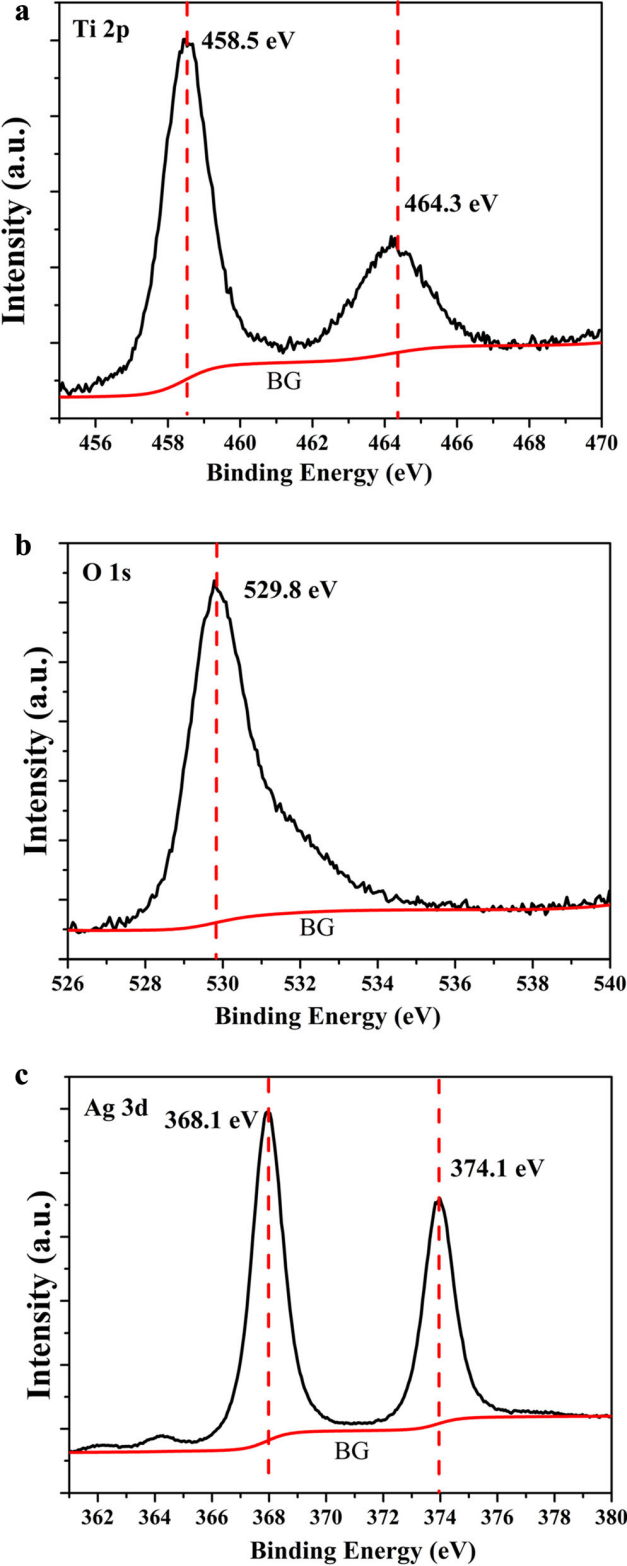
XPS spectra of Ag(30s)/TiO_2_: **a** high-resolution XPS of Ti 2p peaks, **b** high-resolution XPS of O 1s peaks, and **c** high-resolution XPS of Ag 3d peaks

To further demonstrate the successful fabrication of Ag NPs deposited on three-dimensional pine tree-like hierarchical TiO_2_ nanotube arrays, the elemental chemical status and compositions of Ag(30s)/TiO_2_ were analyzed by XPS. Figure 4a shows the XPS spectrum of Ti 2p. It can be observed that two peaks at binding energies of 464.3 and 458.5 eV can be attributed to Ti 2p_3/2_ and Ti 2p_1/2_, respectively. The result demonstrates the state of Ti^4+^ in the anatase TiO_2_ [40]. The O 1s peaks at binding energy of 529.8 eV are attributed to the typical signal of Ti-O-Ti as shown in Fig. 4b [41]. The Ag 3d XPS spectrum shown in Fig. 4c consists of two peaks at 368.1 and 374.1 eV with a distance of approximately 6.0 eV. These binding energies are consistent with Ag 3d_5/2_ and Ag 3d_3/2_, confirming that Ag NPs primarily exist in the metallic form in the Ag/TiO_2_ composite [42].

**Fig. 5:**
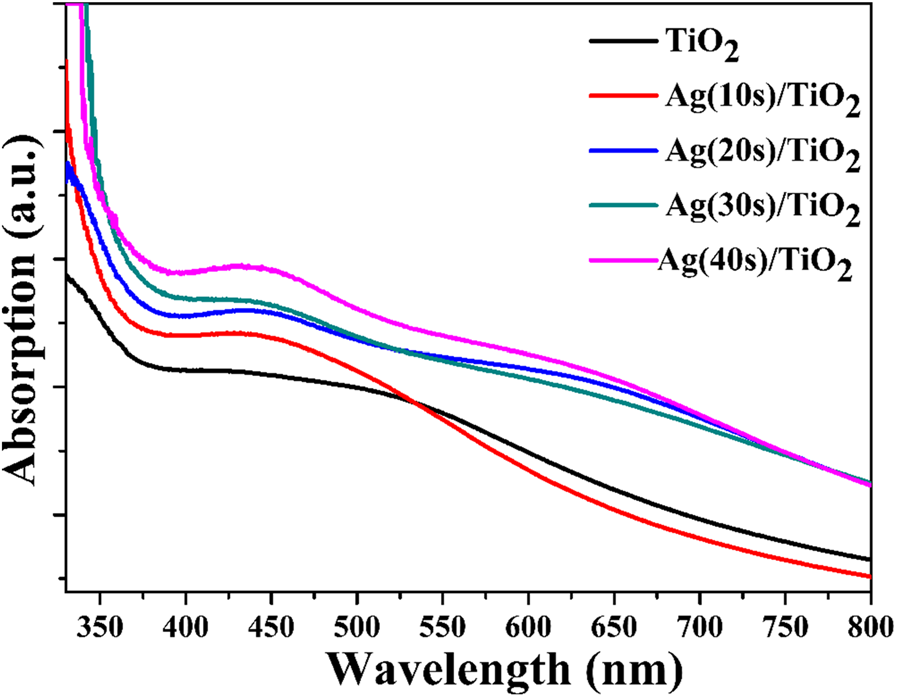
UV-vis absorption spectra of TiO_2_ and Ag/TiO_2_ composite structure with different Ag deposition times

The technique to characterize the plasmonic response absorption of Ag NPs is to investigate the UV-vis absorption spectrum. At Ag LSPR frequency, Ag NPs exhibit strong absorption. Figure 5 displays the absorption spectra of TiO_2_ and Ag NPs deposited on the branches of TiO_2_ with different deposited times. The absorption edge nearby 400 nm belongs to the optical band gap absorption of TiO_2_ [43]. The increased absorption peak at around 425 nm belongs to the SPR of Ag NPs. It also shows that absorption peak increases with the increase of Ag NPs on the surface of TiO_2_. It can be observed that the position of Ag NPs SPR is close to the exciton absorption of TiO_2_. Hence, it is beneficial for the energy coupling of the TiO_2_ plasmonic photocatalyst.

**Fig. 6:**
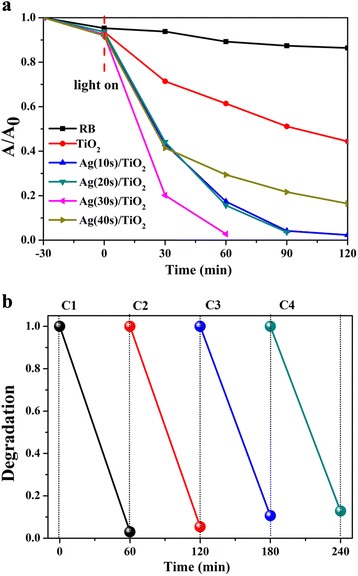
**a** The typical degradation curve of RhB in the presence of TiO_2_ and Ag/TiO_2_ composite structure with different Ag deposition times under UV and visible light irradiation. **b** Degradability of different cycling runs for photocatalytic degradation of RhB of Ag(30s)/TiO_2_ composite

The phototcatalytic activity of TiO_2_ array films, Ag(10s)/TiO_2_, Ag(20s)/TiO_2_, Ag(30s)/TiO_2_, and Ag(40s)/TiO_2_ composite systems with an area about 6 cm^2^ was evaluated by degradation of the rhodamine B (RhB) solution under UV and visible irradiation, and the temperature was maintained at 18 °C in the process of photocatalytic reaction (Table 1). And the RhB solution was also measured under the same experimental conditions. The irradiation time interval is 30 min. As shown in Fig. 6a, the concentration of RhB is decreased upon the irradiation time. The RhB decolorization rate for the three-dimensional pine tree-like hierarchical TiO_2_ nanotube can only approach 55% after 2-h UV-vis light irradiation. However, it is found that the photocatalytic efficiency of the Ag/TiO_2_ composite films increases significantly than the pure TiO_2_. The Ag(30s)/TiO_2_ shows the highest photocatalytic performance that degraded 98% of RhB after the UV-vis light irradiation for 1 h. The degradation of Ag(20s)/TiO_2_ is 96% after 1.5-h UV-vis light irradiation. The degradation of Ag(10s)/TiO_2_ is 98%, and the degradation of Ag(40s)/TiO_2_ is 84% after 2-h UV-vis light irradiation. Moreover, the photocatalytic reaction of semiconductor materials can be accounted for by Langmuir-Hinshelwood (L-H) model, The L-H model equation is as below [44]:

**Table 1:** The parameters on spectral distribution and relative intensity of the used mercury lamp in the photocatalytic tests

Wavelength (nm)	250	313	365	400	510	620	720
Relative intensity (%)	20	85	100	30	20	40	80

$$ \ln \left({A}_0/A\right) = kt $$

where *A*_0_/*A* represents the ratio of concentration of the dye at adsorption-desorption equilibrium and after irradiation for time *t*. And the *k* is the apparent first-order reaction rate constant (min^−1^). The *k* value of TiO_2_ and Ag/TiO_2_ composite structure with different Ag deposition times are shown in Table 2. The *k* value of pure TiO_2_ is 9 × 10^−3^ min^−1^, and the Ag(30s)/TiO_2_ shows that the largest *k* value is about 6 × 10^−2^ min^−1^. The *k* value of Ag(30s)/TiO_2_ is nearly seven times than that of pure TiO_2_, and the *k* value of other samples is 3.7 × 10^−2^ min^−1^(Ag(20s)/TiO_2_), 3.5 × 10^−2^ min^−1^(Ag(10s)/TiO_2_), and 1.5 × 10^−2^ min^−1^ (Ag(40s)/TiO_2_). The enhancement of the photocatalytic efficiency is significant for all Ag deposited samples than the pure TiO_2_, and the photocatalytic efficiency of Ag/TiO_2_ is increased with the increase of Ag deposition time, but with further increase of the deposition time to 40 s, the sample shows lower photocatalytic efficiency than other samples. Therefore, the optimum Ag deposition time is 30 s in the research.

**Table 2:** The degradation of RhB of TiO_2_ and Ag/TiO_2_ composite structure with different Ag deposition times under UV and visible light irradiation

Sample	TiO_2_	Ag(10s)/TiO_2_	Ag(20s)/TiO_2_	Ag(30s)/TiO_2_	Ag(40s)/TiO_2_
Degradation time (min)	120	120	90	60	120
Degradation (%)	55	98	96	98	84
*k* value (min^−1^)	9 × 10^−3^	3.5 × 10^−2^	3.7 × 10^−2^	6 × 10^−2^	1.5 × 10^−2^

The stability of the photocatalyst is very important for practical applications. Therefore, the stability of Ag(30s)/TiO_2_ has been further evaluated by recycling the photocatalyst for RhB degradation as shown in Fig. 6b. It can be seen that the degradation rate of RhB solution is more than 90% after 4 cycles with duration of 60 min per cycle, which is to say that the photocatalytic efficiency of Ag(30s)/TiO_2_ does not exhibit obvious loss after several recycles. The result shows that the Ag/TiO_2_ composite has high stability during the photocatalytic degradation of RhB.

On the basis of the experiment results, the outstanding photocatalytic performance of Ag/TiO_2_ may be explained as follows:

1. Large specific surface area and fast charge transport.
  Three-dimensional pine tree-like hierarchical TiO_2_ nanotube arrays composing of a vertically oriented nanotube trunks and grafted with large amounts of short branches that have a large surface and enhance the absorption of dye molecules. Moreover, the photo-induced electrons directly transport through the nanotube [45], and the nanotube provides efficient transport pathway for rapid charge transport that leads to improving the electron collection and the electron-hole separation. Therefore, the photocatalytic activity of TiO_2_ could be increased.
2. LSPR-mediated local field enhancement.
  It is well known that the LSPR effect of Ag NPs can induce a strong local electric field. Moreover, here, a large number of Ag NPs are uniformly located on the branches of TiO_2_, so near-field dipolar interactions between adjacent particles were very strong. Therefore, the LSPR of Ag NPs can enhance the local field near the surface of NPs as well as the giant field enhancement between adjacent particles [39, 46, 47]. Hence, there may be induced strong local electric field in the structure. The strong local electric field can increase the light capturing, and therefore boosts the generation of electron-hole pairs in Ag/TiO_2_ composite, and hence improves the performance of photocatalysis. The typical Raman spectra of TiO_2_, Ag(10s)/TiO_2_, Ag(20s)/TiO_2_, Ag(30s)/TiO_2_, and Ag(40s)/TiO_2_ are shown in Fig. 7. It can be observed that the Raman intensity of Ag/TiO_2_ composites increases compared to that of pure TiO_2_. The Raman scattering intensity increased with the increase of Ag deposition time and then decreased when the deposition time reaches 40 s, and the sample of Ag(30s)/TiO_2_ shows the strongest Raman intensity. As is well known that Raman scattering intensity is proportional to the square of the intensity of a local field [47], Raman peak intensity enhancement is due to LSPR-mediated large near-field enhancement. The scattering and absorption cross-sections are separately proportional to R^6^ and R^3^ when the size of NPs is much smaller than the wavelength of light. As a result, for small particles (about <30 nm), the optical response dominates by absorption. Moreover, the absorption increases with the increased size of Ag NPs, it implies that the local electrical field induced by LSPR will increase with the increase of NP size. Therefore, the Ag(30s)/TiO_2_ shows high enhancement factors of Raman scattering. However, the optical response dominates by scattering for larger particles [48, 49], so, the local field induced by LSPR of the Ag NPs of Ag(40s)/TiO_2_ is weaker than that of other samples. On the other hand, due to the light scatter increase for Ag(40s)/TiO_2_, the optical path increases, so the absorption of light can also increase. Therefore the Ag(40s)/TiO_2_ also shows much higher photocatalytic performance than that of pure TiO_2_. Based on our experiment results and literature, the boost generation of the electron-hole pairs due to the effect of LSPR is the critical factor to enhance the photocatalytic activity of TiO_2_.

**Fig. 7:**
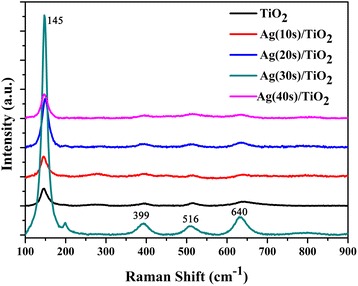
Raman spectra of TiO_2_ and Ag/TiO_2_ composite structure with different Ag deposition times
3. The electron transfer from Ag to TiO_2_.
  Figure 1c illustrates the possible charge transfer process in Ag/TiO_2_ system. When TiO_2_ is irradiated by light with energy higher than its band gap, valence electron is excited into the conduction band and hole still in the valence. Valence holes accumulated to the surface and induced the surface hydroxyl radical · OH [50], then oxidated the decomposition of RhB. However, the generated electrons would transfer from the TiO_2_ to Ag NPs because the work function of TiO_2_ (4.2 eV [51]) is lower than that of Ag (4.52 to 4.74 eV [52]), and Ag NPs act as electron trap which effectively facilitate the separation of photogenerated carriers, thus improves the transfer efficiency of electron and hole pairs [53, 54]. The electrons on Ag NPs will be transferred to the absorbed oxygen and form superoxide, the formed superoxide is responsible for the reduction of organic RhB [55]. Thus, the Ag/TiO_2_ structure can efficiently prevent the recombination of electron and hole, therefore improves the photocatalytic efficiency of TiO_2_.

## Conclusions

In summary, the high-performance plasmonic photocatalyst three-dimensional pine tree-like hierarchical TiO_2_ nanotube array films loaded with Ag NPs were fabricated by a simple two-step process. A large number of uniform Ag NPs dispersed in the pin tree-like hierachical TiO_2_, which effectively improved the light harvest, boosted the generation of electron and hole pairs, and notably improved the separation, transport, and electron-hole pairs with large specific surface area, significantly improved the photocatalytic performance under UV-visible light irradiation (seven times than pure TiO_2_). Therefore, this research supplies an effective synthetic strategy for noble metal NP-modified three-dimensional hierarchical TiO_2_, which will be of great significance for promising applications in the fields of environment and energy for high-efficiency light-energy conversion.

## Methods

### Synthesis of Three-Dimensional Pine Tree-Like Hierarchical TiO_2_ Nanotube Array Films

Three-dimensional tree-like TiO_2_nanotube arrays were synthesized using a hydrothermal method. The details of the synthetic procedure were described by Roh et al. [56]. Briefly, 0.73 g of potassium titanium oxide oxalate dehydrates (PTO) was dissolved in 7-ml deionized water, and then, the mixed solution was added to 33-ml diethylene glycol (DEG) and stirred well. Fluorine-doped tin oxide (FTO) transparent conductive glass substrates were washed by isopropanol, chloroform, and deionized water successively. And then, a cleaned FTO was placed in a teflon-lined stainless steel autoclave filled with mixed solutions. The hydrothermal reactions temperature is 200 °C, and the reaction time is 11 h. After the reaction, the as-synthesized samples were washed with water more than once. After that, the as-prepared samples were annealed in air at 500 °C for 1 h to remove the residuary organic substance.

### Preparation of Ag/TiO_2_ Composites

Ag NPs were deposited on three-dimensional pine tree-like hierarchical TiO_2_ nanotube array films by a magnetron sputtering system. The deposition rate of Ag is about 20 nm min^−1^, and deposition time is 10, 20, 30, and 40 s, then obtained Ag NP-decorated three-dimensional pine tree-like hierarchical TiO_2_ nanotube array films were named as Ag(10s)/TiO_2_, Ag(20s)/TiO_2_, Ag(30s)/TiO_2_, and Ag(40s)/TiO_2_.

### Characterization

The morphology and microstructure of the sample was examined by SEM (S4800, Hitachi) operated at an acceleration voltage of 5 kV. The crystallinity and phase constitutions of all samples were analyzed with X-ray diffraction (XRD) (D8 Germany, Bruker Axs). The elemental chemical status and compositions were analyzed with X-ray photoelectron spectroscopy (XPS) using Mg Ka1, 2 (1253.6 eV) excitation. The Raman scattering spectra were analyzed by a micro-Raman system (LabRAM HR800, HORIBA JobinYvon, Paris, France). Ar laser (532.0 nm) is the excitation source, and laser power was kept at 2.5 mW. The UV-vis absorption spectra of all samples were determined by UV-visible dual-beam spectrophotometer (Shimadzu UV 2550).

### Photocatalytic Activity Measurement

The photocatalytic activity of the prepared sample with an area of about 6 cm^2^ was evaluated by decolorization of 10-ml rhodamine B (RhB) solution with the concentration of 10 mg/L. Mercury lamplight as a UV and visible light source (spectral distribution and relative intensity of the mercury lamp in Table 1). The temperature was maintained at 18 °C in the process of photocatalytic reaction by equipping with a water circulation facility. Before irradiation, all samples were put into 10-ml RhB solution for 30 min in darkness in order to establish an adsorption/desorption equilibrium of RhB molecules on the surface of the photocatalysts. The degradation of RhB solution was determined by using an UV-vis spectrophotometer (Shimadzu UV 2550) at 554.0 nm. The time interval is 30 min, and the total reaction time was 2 h. As a comparison, the RhB solution was also measured under the same experimental conditions.

## Abbreviations

D: Dimensional
DEG: Diethylene glycol
FTO: Fluorine-doped tin oxide
LSPR: Localized surface plasmon resonance
NPs: Nanoparticles
PTO: Potassium titanium oxide oxalate dehydrates
RhB: Rhodamine B
SEM: Scanning electron microscopy
XPS: X-ray photoelectron spectroscopy
XRD: X-ray diffraction
